# Left Atrial Stiffness Increases after Trans-Catheter Atrial Septal Closure

**DOI:** 10.3390/jcm13020327

**Published:** 2024-01-06

**Authors:** Shuhei Tanaka, Teruhiko Imamura, Nobuyuki Fukuda, Hiroshi Ueno, Koichiro Kinugawa

**Affiliations:** Second Department of Internal Medicine, University of Toyama, Toyama 930-0194, Japan; stanaka@med.u-toyama.ac.jp (S.T.); nfukuda@med.u-toyama.ac.jp (N.F.); hiroshi.ueno.md@gmail.com (H.U.); kinugawa0422@gmail.com (K.K.)

**Keywords:** atrial septal defect, transcatheter atrial septal closure, left atrial stiffness

## Abstract

Background: Transcatheter atrial septal closures for secundum atrial septal defects (ASD) have demonstrated favorable clinical outcomes. However, the impact of device implantation on the stiffness of the left atrium remains unclear. Method: Patients with secundum ASD undergoing transcatheter closure and follow-up right heart catheterization at six months were included. We investigated the relationship between post-procedural (E/e’ ratio)/(LAs strain) ratio, an index of left atrial stiffness, and baseline characteristics, including echocardiographic and hemodynamic parameters. Results: Forty patients were included (median 69 (56, 75) years, 12 men, and pulmonary systemic flow ratio 2.27 (1.96, 2.86)). Trans-catheter ASD closure was successfully performed without any major complications, accompanying a significant reduction in right ventricular to left ventricular size ratio from 1.04 (0.87, 1.13) to 0.74 (0.66, 0.86) (*p* < 0.01). The (E/e’ ratio)/(LAs strain) ratio was markedly elevated the day after the procedure and was further increased 6 months later (before: 0.25 (0.17, 0.34), 1 day later: 0.34 (0.27, 0.50), 6 months later: 0.43 (0.27, 0.76), *p* < 0.01). The groups with higher (E/e’ ratio)/(LAs strain) ratios at 6 months had significantly more severe heart failure conditions including lower cardiac output and higher plasma B-type natriuretic peptides. Conclusions: Patients undergoing transcatheter ASD closure experienced improvement in hemodynamics and clinical symptoms but an elevation in left atrial stiffness post-procedure. The clinical ramifications of this finding, particularly during the longer-term observation period subsequent to ASD closure, warrant further investigation.

## 1. Introduction

Atrial septal defect (ASD) is the most common congenital heart disease, accounting for nearly 40% of all congenital heart diseases [1]. The majority of pediatric patients with ASD are asymptomatic and often undiagnosed. Such patients may be diagnosed in adulthood incidentally or with the onset of symptoms, such as supraventricular arrhythmias and heart failure. Of note, the mortality of untreated patients with ASDs may be as high as 90% at 60 years of age [2]. This is a rationale that supports contemporary indication of ASD closure so far.

A trans-catheter closure of secundum ASD has been established as an alternative to surgical procedures with non-inferior efficacy and feasibility [3]. However, long-term prognosis after trans-catheter closure of secundum ASD is controversial [4]. Hemodynamics after trans-catheter closure of secundum ASD may be key to further understanding its clinical impact.

Recently, the concept of “left atrial (LA) stiffness” has been proposed to understand the diastolic dysfunction of the LA [5]. Stiff LA syndrome is representative of increased LA stiffness due to elevated LA pressure and successive pulmonary hypertension and right heart failure [6]. Stiff LA syndrome is encountered at the time when the LA is directly or indirectly affected by any interventions, including mitral valve replacement and catheter ablation for atrial fibrillation [7]. Stiff LA syndrome is associated with poor prognosis in patients with preserved ejection fraction [8].

Theoretically, trans-catheter closure of secundum ASD may also increase LA pressure by the loss of the left–right shunt. We hypothesized that such a procedure may cause stiff LA syndrome in some patients and those with such a syndrome may have worse clinical outcomes. In addition, there are few reports of invasive hemodynamic assessment in the chronic phase post-ASD closure. This retrospective study evaluated the association between LA stiffness and clinical variables including hemodynamic parameters post-ASD closure and further investigated the prognostic impact of post-procedural LA stiffness.

## 2. Materials and Methods

### 2.1. Patient Selection

Consecutive patients with secundum ASD, who underwent trans-catheter ASD closure at a solitary academic institution within the time frame spanning June 2016 to September 2021, were deemed eligible for inclusion in this retrospective investigation. Inclusion criteria encompassed patients aged 40 years and above, individuals who underwent right heart catheterization at the six-month post-procedure interval, and those who underwent transthoracic echocardiography (TTE) during the six-month follow-up period.

This study was conducted in strict accordance with the principles outlined in the Declaration of Helsinki and adhered to the ethical standards established by the responsible committee overseeing human experimentation. All participants provided written informed consent prior to their involvement in the study, and the study protocol received approval from the local ethical committee.

### 2.2. Indication of Trans-Catheter ASD Closure

Patients diagnosed with secundum ASD exhibiting right heart overload were referred to our institution to evaluate the feasibility of trans-catheter ASD closure. Comprehensive imaging studies were undertaken to evaluate the anatomical suitability for trans-catheter ASD closure, encompassing assessments of defect size and the adjacent rim dimensions. Additionally, eligible patients were required to maintain a preserved left ventricular ejection fraction. Furthermore, individuals with pulmonary hypertension characterized by a pulmonary vascular resistance exceeding 3.0 wood units were deemed ineligible for the procedure. Lastly, patients were disqualified from consideration if they presented with any additional anatomical abnormalities apart from the ASD.

### 2.3. The Procedure of Trans-Catheter ASD Closure

All patients underwent trans-catheter ASD closure through a trans-femoral vein approach while under general anesthesia, following established procedural protocols. A guidewire was passed through the defect, and a balloon closure test was subsequently conducted. Following precise measurement of the defect, the appropriate device type and size were meticulously selected and subsequently implanted. Two distinct device models were employed in this procedure: the Amplatzer septal occluder (Abbott Vascular, IL, USA) and the Figulla Flex II (Occlutech GmbH, Helsingborg, Sweden). Post-device implantation, meticulous confirmation ensured that the device encompassed the atrial septum entirely by transesophageal echocardiography and the wiggle test, which pushed and pulled the delivery cable to ensure that the device did not dislodge before it was detached from the delivery cable. Postoperatively, the patient underwent antithrombotic therapy for approximately six months.

### 2.4. Data Collection

Preoperative baseline characteristics including demographics, TTE findings, and hemodynamic data were collected. Epiq 7G or CVx (Philips Healthcare, Amsterdam, The Netherlands) were used for TTE.

Postoperative data, except for hemodynamic parameters, were collected the day after ASD closure. The mid-term follow-up data, including hemodynamic parameters, were collected 6 months after the intervention. Cardiovascular events including cardiac death or heart failure readmissions were counted for two years or until June 2023 following the index ASD closure, which was defined as day 0.

Additional echocardiographic analysis including LA peak systolic (LAs) strain was performed offline using QLAB workstation (Philips Healthcare, Amsterdam, The Netherlands) in a blinded manner to the study data. Researchers who measured LA strain were blinded to all clinical data at the time of data measurements. In sinus rhythm, the data were recorded in 3 consecutive heartbeats, and in atrial fibrillation, in 5 consecutive heartbeats. Global LA strain measurements were taken from 4 atrial walls from apical 4-chamber images and 2-chamber images at end-expiration and averaged over 3 consecutive cardiac cycles. Segments were excluded if signal quality was poor.

### 2.5. LA Stiffness

**Figure 1:**
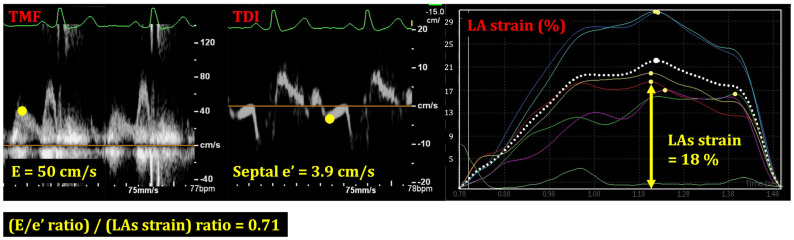
How to measure (E/e’ ratio)/(LAs strain) ratio. LAs strain refers to LA strain during the systolic phase of cardiac cycle. The measurement depicted in this figure assesses the strain on the septal side of the LA. The other lines illustrate strains in various parts of the LA. TMF, trans-mitral flow; TDI, tissue Doppler imaging; LA, left atrium.

LA stiffness was defined as an increase in LA pressure induced by passive filling divided by the increase in LA volume [5]. Mitral E/e’ ratio reflects left ventricular (LV) filling pressure because it is a measure of the pressure gradient between LV and LA in early diastole. LAs strain is a measure of the maximum rate of change in the long axis of the LA wall and represents the rate of volume change in passive filling. Thus, LA stiffness was assessed by the index: (E/e’ ratio)/(LAs strain) ratio (Figure 1). A previous study reported an average value of (E/e’ ratio)/(LAs strain) ratio as 0.9 in patients with diastolic dysfunction [5]. We used this value as a cutoff in this study.

### 2.6. Statistical Analyses

The principal objective of this study was to delineate the trajectory of the (E/e’ ratio)/(LAs strain) ratio over the six-month observation period following trans-catheter ASD closure. Statistical analyses were conducted employing JMP Pro 16 (SAS Institute Inc., Cary, NC, USA). Significance was determined based on two-tailed *p*-values below 0.05. Continuous variables were presented as medians with corresponding interquartile ranges, while categorical variables were expressed in terms of numerical counts and percentages.

The Wilcoxon-signed rank test was employed to compare paired data. The patient cohort was dichotomized into two groups using a threshold of the (E/e’ ratio)/(LAs strain) ratio derived from the six-month follow-up assessment: 0.9.

## 3. Results

### 3.1. Baseline Characteristics

The baseline characteristics of 40 patients are displayed in Table 1. The median age was 69 (56, 75) years and 33% were male patients. Patients with atrial fibrillation accounted for 28% of all participants. Most of them (73%) had a history of catheter ablation. Most patients had New York Heart Association (NYHA) class I or II and 10% had NYHA class III. The plasma B-type natriuretic peptide (BNP) level was 42 (17, 121) pg/mL. The serum creatinine level was 0.71 (0.61, 0.86) mg/dL. The mean pulmonary artery pressure (PAP) was 19 (14, 23) mmHg and the pulmonary artery wedge pressure (PAWP) was 9 (7, 11) mmHg. The mean pulmonary-to-systemic flow ratio (Qp/Qs) was 2.27, indicating significant right heart volume overloading.

### 3.2. Baseline Echocardiography Data

The LV end-diastolic diameter was 39 (37, 43) mm and the LV ejection fraction (LVEF) was 66% (59%, 73%) (Table 1). The right ventricle (RV) was enlarged with an RV end-diastolic diameter of 41 (37, 55) mm and an RV/LV ratio of 1.05 (0.88, 1.13). The RV fractional area change and tricuspid annular plane excursion (TAPSE) were within normal range in many patients. The (E/e’ ratio)/(LA strain) ratio, which was a primary concern of this study, was 0.24 (0.17, 0.32) median, which was similar to the healthy cohort.

The median defect size was 11 (9, 15) mm in the short diameter and 17 (13, 22) mm in the long diameter, respectively, except for two patients with double defects. Seventeen patients (43%) exhibited rim defects, defined as a length of rim measuring under 5 mm.

### 3.3. Procedure Data

**Figure 2:**
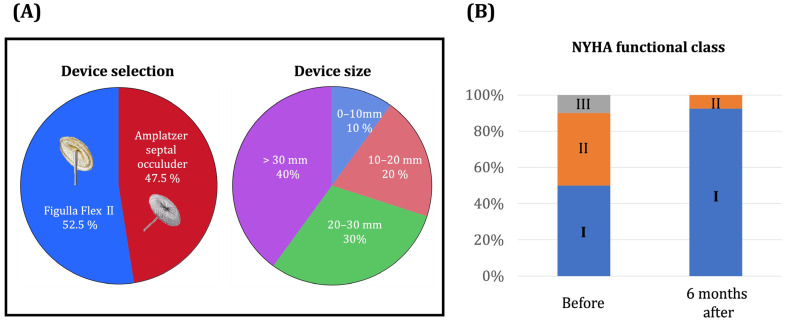
(**A**) Selections of device type and size for trans-catheter ASD closure. (**B**) Changes in symptoms following trans-catheter ASD closure. There was no bias in the type or size of devices used. And heart failure symptoms clearly improved after treatment. I–III, NYHA functional class I–III.

The majority of patients (with the exception of a single individual who necessitated the implantation of two devices due to multiple defects) received a singular device. Approximately half of the patients (52.5%) were fitted with the Figulla Flex II device, while the remaining patients received the Amplatzer septal occluder (Figure 2). A substantial proportion, roughly 70% of patients, received devices with sizes exceeding 20 mm. All patients underwent successful ASD closure without any peri-procedural complications.

### 3.4. Post-Procedure Data

All patients underwent the procedure successfully without any procedure-related complications. Post-procedure, the NYHA functional class significantly improved (*p* < 0.01) and no patients had NYHA class III or IV. No patients had heart failure hospitalization or cardiac death during the 2-year observational period after the procedure. Plasma BNP tended to decrease at 6 months follow-up.

**Figure 3:**
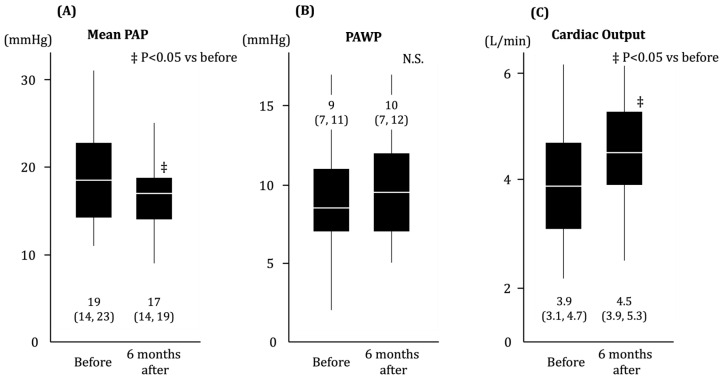
Change in hemodynamics following trans-catheter ASD closure. (**A**) Mean PAP, (**B**) PAWP, (**C**) Cardiac output. ‡ *p* < 0.05 by Wilcoxon signed-rank test. In the mid-term follow up, there was an increase in cardiac output without a deterioration in LA pressure. PAP, pulmonary artery pressure; PAWP, pulmonary artery wedge pressure; N.S., no significant difference.

Hemodynamics changes during the 6 month observation period are displayed in Figure 3. The mean PAP levels further decreased within normal range (*p* < 0.01). PAWP remained unchanged (*p* = 0.27). Cardiac output increased significantly (*p* < 0.01).

**Figure 4:**
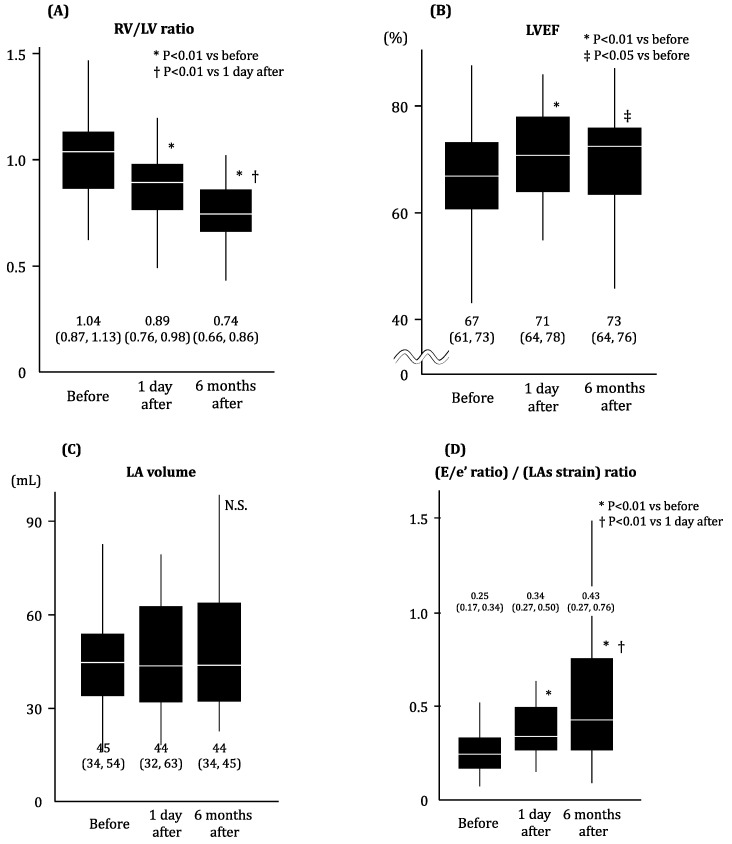
Change in echocardiographic parameters following trans-catheter ASD closure. (**A**) RV/LV ratio, (**B**) LVEF, (**C**) LA volume, (**D**) (E/e’ ratio)/(LAs strain) ratio. * *p* < 0.01 vs. before the intervention by Wilcoxon signed-rank test, † *p* < 0.01 vs. 1 day after the intervention by Wilcoxon signed-rank test, ‡ *p* < 0.05 vs. before the intervention by Wilcoxon signed-rank test. In the mid-term follow-up, the RV/LV ratio exhibited continuous improvement from the day after the procedure, whereas the (E/e’ ratio)/(LAs strain) ratio remained elevated from the day after the procedure. RV, right ventricular; LV, left ventricular; EF, ejection fraction; LA, left atrium; N.S., no significant difference.

Trends in TTE during the 6-month observation period after ASD closure are displayed in Figure 4. The RV/LV ratio decreased significantly immediately at day 1 and further decreased 6 months later (*p* < 0.05 for both; Figure 4A). LVEF increased immediately at day 1 (*p* < 0.01) and remained unchanged at the 6-month follow-up (*p* > 0.05; Figure 4B). The LA volume remained unchanged (*p* > 0.05; Figure 4C). The (E/e’ ratio)/(LA strain) ratio, which was an index of LA stiffness and a primary concern of this study, increased immediately after the procedure and further increased at the 6-month follow-up (*p* < 0.05 for both; Figure 4D). Moreover, patients with NYHA functional class II following ASD closure had a significantly higher (E/e’ ratio)/(LA strain) ratio than patients with NYHA functional class I following this procedure (*p* = 0.01).

### 3.5. Predictors for the High LA Stiffness at 6-Month Follow-Up

The study endpoint was defined as an (E/e’ ratio)/(LAs strain) ratio > 0.90, which was the median value of the diastolic dysfunction group in the previous literature [5]. In total, seven patients had an (E/e’ ratio)/(LAs strain) ratio > 0.90 at 6-month follow-up.

Several baseline characteristics were associated with an (E/e’ ratio)/(LAs strain) ratio > 0.90, including plasma BNP (odds ratio per 10 pg/mL 1.35, 95% confidence interval 1.11–1.65, *p* = 0.0028), TAPSE (odds ratio per 1 mm 0.76, 95% confidence interval 0.64–0.93, *p* = 0.0074), and Qp/Qs (odds ratio per 0.1 7.03, 95% confidence interval 1.72–28.81, *p* = 0.0067) (Table 2). The cutoff value of BNP, TAPSE, and Qp/Qs to satisfy the endpoint was calculated as 134.7 pg/mL, 23.2 mm, and 2.76 with an area under the curve of 0.948, 0.857, and 0.870, respectively.

## 4. Discussion

In this investigation, our focus lay in the assessment of alterations in hemodynamic parameters and echocardiographic data, including LA stiffness, during the six months subsequent to trans-catheter ASD closure. Our findings revealed that the (E/e’ ratio)/(LAs strain) ratio, a metric indicative of LA stiffness, exhibited an increase following trans-catheter ASD closure. In contrast, hemodynamic parameters and plasma BNP levels displayed improvement at the six-month post-intervention mark. Baseline factors, namely elevated BNP levels, reduced TAPSE, and an increased Qp/Qs, emerged as significant predictors for elevated LA stiffness at the six-month follow-up.

### 4.1. The Efficacy of Trans-Catheter ASD Closure

Trans-catheter ASD closure is less invasive than surgical ASD repair and its efficacy is reported as comparable to the surgery [3]. There are some unsuitable morphologies for trans-catheter ASD closure including insufficient surrounding rims, large defects, and multiple defects [9]. However, the evolution of devices and techniques have made it possible to undergo some unsuitable cases by trans-catheter. For instance, only the aortic rim deficiency may be overcome by implanting flare to the aortic root [10]. Multiple defects may be treated with one or two devices with caution of rims and intracardiac structures around the septum [11].

While a relatively large number of patients have minor symptoms, their symptoms improve immediately following the procedure [12]. In echocardiographic response to ASD closure, right ventricular reverse remodeling was obvious immediately the day after the procedure and the trend was maintained [13]. This trend is associated with a younger age at the treatment and a less degree of right ventricular dilatation before the repair [14]. On the other hand, in some adult patients with pre-existing decreased left ventricular compliance, the increase in preload due to ASD closure can cause elevation of LA pressure and lung congestion [15,16].

In this study, we performed hemodynamic evaluation in the chronic phase using an invasive method that has rarely been reported. On the whole, the mean PAP decreased and cardiac output increased markedly. There was no significant change in PAWP, reflecting left atrial pressure, following ASD closure. However, to varying degrees, PAWP was elevated following ASD closure in 40% of all cases in this study. Although events were difficult to detect during the current observation period, these results are sufficient to indicate that elevated LA pressure should be a concern in some cases.

### 4.2. Clinical Implication of LA Stiffness following Trans-Catheter ASD Closure

The mechanism of stiff LA syndrome includes the fibrosis of atrial muscle. In cases after trans-catheter ASD closure, it is assumed that the implantation of a metal device in the septum will restrict the mobility of the atrium. In this study, the (E/e’ ratio)/(LAs strain) ratio was elevated in 93% of all cases 6 months after ASD closure, suggesting a worsening of LA stiffness after device implantation. Factors associated with higher measurement of LA stiffness after device implantation included PAWP and BNP, as expected, but were also associated with Qp/Qs, pulmonary artery pressure, and right atrial pressure, which generally suggest right heart overload. In the group with a higher (E/e’ ratio)/(LAs strain) ratio after correction of ASD, preoperative LA pressure was significantly higher but still relatively low. The left-to-right shunt through the ASD may have buffered the elevated left atrial pressure and masked potential left-sided heart failure, which clinically is actualized after ASD closure and the loss of left-to-right shunt.

As a clinical application of this logic, an interatrial shunt device has recently been developed for heart failure, especially heart failure with preserved ejection fraction [17]. There is no proven evidence that it inhibits cardiovascular events, but it has been shown to increase exercise tolerance and inhibit the elevation of LA pressure during exercise [18]. Further investigation may reveal the optimal strategy of not closing the ASD based on the degree of left-sided heart failure and the size of the ASD.

Recently, the (E/e’ ratio)/(LAs strain) ratio, which reflects LA stiffness, has been reported to be a predictor of cardiovascular events [8]. Its observation period is very long, and its impact may not be significant in the short term. Certainly, our study also demonstrated improved clinical findings after trans-catheter ASD closure, except for incremental LA stiffness. However, it should be noted that in the long term, an increase in LA stiffness may impact the occurrence of heart failure. We do not deny at all the current indication for percutaneous ASD closure according to favorable clinical outcomes including outcomes. Nevertheless, follow-up after ASD closure is highly recommended to be continued, particularly in patients with elevated baseline LA stiffness, as long as possible.

### 4.3. Limitations

Several limitations of the present study should be addressed. First, this is a retrospective single-center analysis among a small-size cohort. Statistical non-significance in this study may reach statistical significance in the larger-size studies. Second, this study includes patients with atrial fibrillation. In patients with atrial fibrillation, the LA contraction is poor and may have less clinical significance because LA strain reflects the contractile force of longitudinal. Nevertheless, there should still be clinical significance in observing trajectory such as hemodynamics and morphology following ASD closure. Third, because of the short observation period, cardiovascular events could not be detected, and their prognostic impact could not be assessed sufficiently.

## 5. Conclusions

After trans-catheter ASD closure, symptoms and clinical findings indicating right heart volume overload significantly improved six months later. This is a strong rationale to perform ASD closure according to the contemporary indication. In contrast, the LA stiffness worsened from the day after the procedure and became more pronounced six months later. Theoretically, such an incremental LA stiffness may have some unknown negative prognostic impact. Further longer-term investigation is needed to clarify its prognostic impact and construct a strategy for optimal patient selection.

## Figures and Tables

**Table 1:** Baseline characteristics.

*N*	40
Demographics	
Age, years	69 (56, 75)
Male, *n* (%)	12 (30)
Body surface area, m^2^	1.50 (1.43, 1.69)
NYHA class I/II/III/IV	20/16/4/0
AF, *n* (%)	10 (25)
History of AF ablation, *n* (%)	8 (20)
Hypertension, *n* (%)	16 (40)
Diabetes mellitus, *n* (%)	4 (10)
Ischemic heart disease, *n* (%)	2 (5)
Medication	
Loop diuretics, *n* (%)	10 (25)
RAS inhibitor, *n* (%)	13 (33)
β blocker, *n* (%)	10 (25)
Calcium channel blocker, *n* (%)	13 (33)
Laboratory data	
Serum albumin, g/dL	4.2 (4.0, 4.5)
Serum creatinine, mg/dL	0.70 (0.61, 0.85)
Serum total-bilirubin, mg/dL	0.7 (0.6, 0.9)
Hemoglobin, g/dL	13.2 (12.7, 14.3)
Plasma BNP, pg/mL	36.1 (16.7, 121.2)
Hemodynamic	
Systolic blood pressure, mmHg	129 (108, 138)
Diastolic blood pressure, mmHg	70 (64, 77)
Heart rate, bpm	70 (63, 75)
Mean PAP, mmHg	19 (14, 23)
PAWP, mmHg	9 (7, 11)
RAP, mmHg	6 (4, 9)
Qp/Qs	2.27 (1.96, 2.86)
PVR, wood·unit	1.08 (0.74, 1.66)
Echocardiography	
LA volume, mL	45 (34, 54)
LV end-diastolic dimension, mm	39 (37, 43)
LV end-systolic dimension, mm	24 (23, 29)
LV ejection fraction, %	67 (61, 73)
Trans-mitral flow	
E, cm/s	62 (54, 71)
DcT, ms	194 (160, 233)
e’ (average), cm/s	8.2 (6.7, 10.1)
E/e’	7.4 (5.8, 8.3)
RV end-diastolic dimension, mm	41 (37, 44)
TAPSE, mm	26 (20, 29)
RV fractional area change, %	49 (41, 55)
RV/LV ratio	1.04 (0.87, 1.13)
LAs (average) strain, %	31 (23, 40)
E/e’ ratio/LAs strain	0.25 (0.17, 0.34)

**Table 2:** Predictors for the primary outcome: (E/e’ ratio)/(Las strain) ratio > 0.90 at 6-month follow-up.

	Odds Ratio	95% Confidence Interval	*p* Value
Demographic data			
Age, per 1 year	1.37	1.06–1.77	0.0166
Diabetes	6.20	0.70–54.61	0.1003
Hypertension	2.33	0.45–12.23	0.3161
Atrial fibrillation	6.00	1.05–34.1	0.0421
NYHA, per 1 degree	4.38	1.14–16.8	0.0310
Dose of loop diuretics, per 20 mg	4.50	1.31–15.42	0.0167
Hemoglobin, per 1 g/dL	0.41	0.20–0.86	0.0174
Plasma BNP, per 1 pg/mL	1.35	1.11–1.65	0.0028
Echocardiographic data			
LA volume, per 1 mL	1.06	1.01–1.11	0.0131
LV end-diastolic dimension, per 1 mm	0.85	0.69–1.05	0.1376
LV ejection fraction, per 1%	0.99	0.90–1.09	0.8385
RV end-diastolic dimension, per 1 mm	1.20	0.96–1.45	0.0564
RV/LV ratio, per 1	2.49	1.14–5.46	0.0216
TAPSE, per 1 mm	0.76	0.64–0.93	0.0074
ASD long diameter, per 1 mm	1.22	1.02–1.46	0.0273
Hemodynamic data			
Qp, per 1 L/min	1.09	0.81–1.47	0.5715
Qs, per 1 L/min	0.19	0.04–0.78	0.0218
Qp/Qs, per 0.1	7.03	1.72–28.81	0.0067
Mean BP, per 1 mmHg	0.98	0.93–1.03	0.3888
Mean PAP, per 1 mmHg	1.29	1.04–1.59	0.0215
Mean PAWP, per 1 mmHg	1.29	1.00–1.65	0.0460
Mean RAP, per 1 mmHg	1.51	1.06–2.17	0.0238
Others			
Device size, per 1 mm	1.08	0.94–1.23	0.2709

## Data Availability

Data are available from the corresponding authors upon reasonable requests.
